# Challenging behavior in mucopolysaccharidoses types I–III and day-to-day coping strategies: a cross sectional explorative study

**DOI:** 10.1186/s13023-020-01548-9

**Published:** 2020-10-02

**Authors:** Frederik Hoffmann, Susanne Hoffmann, Kevin Kunzmann, Markus Ries

**Affiliations:** 1 Center for Pediatric and Adolescent Medicine, St. Annastift-Hospital Ludwigshafen am Rhein, Karolina-Burger-Straße 5, 67065 Ludwigshafen am Rhein, Germany; 2 Center for Psychiatry, PZN Wiesloch, Heidelberger Straße 1a, Wiesloch, 69168 Germany; 3 Institute of Medical Biometry and Informatics, University of Heidelberg, Im Neuenheimer Feld 347, Heidelberg, 69120 Germany; 4 Pediatric Neurology and Metabolic Medicine, Center for Rare Diseases, Center for Pediatric and Adolescent Medicine, Heidelberg University Hospital, Im Neuenheimer Feld 430, Heidelberg, 69120 Germany

**Keywords:** Mucopolysaccharidoses, MPS, Hurler, Hunter, Sanfilippo, Challenging behavior, Coping, Day-to-day coping

## Abstract

**Background:**

Challenging behavior represents a core symptom in neuropathological mucopolysaccharidoses (MPS) and puts major strain on affected families. Although multimodal approaches including behavioral strategies to treatment could be valuable, there is lack of research to the effectiveness of specific measures. This explorative, cross-sectional study is aimed at the collection of parental experiences regarding effective day-to-day measures against challenging behavior in MPS and focuses on 4 major research questions: First: What is challenging behavior in MPS? Second: Which strategies are helpful in the day-to-day coping with challenging behavior? Third: How strong is parental acceptance of illness and the disorder’s impact on family relationships? Fourth: What are beneficial personal and interfamilial strategies for generally coping with the disorder?

**Methods:**

A semi structured questionnaire was designed de novo in cooperation with affected families. 37/268 questionnaires were returned (rate: 13.8%), of which 34 (MPS I: n = 8, MPS II: n = 8; MPS III: n = 18) could be included in data analysis in accordance with inclusion criteria. Assessment of challenging symptoms was based on perceived frequency, parent- and child stress. Exploration of possible coping strategies for challenging behavior and general illness-related strain included the evaluation of perceived effectiveness. Questionnaires were completed by patient’s relatives and analyzed for strategies to cope with challenging behavior and the disorder’s impact. STROBE criteria were respected.

**Results:**

MPS I was reported to show lower frequency and better perceived manageability of challenging behavior than MPS II and -III. Sleep disturbance, hyperactivity, agitation, aggression and orality seemed relevant symptoms regarding frequency and/or parent stress. Reported measures were manifold, worthwhile approaches against challenging behavior appeared to be aiming at distraction, relief and environmental changes. Medication and non-medication approaches were rated similarly effective. Social exchange, private space and networking with other affected families seemed highly important for personal and interfamilial well-being.

**Conclusions:**

Multimodal mentoring for affected families could be based on the following equivalent pillars: (1) Medication therapy for challenging behavior including evaluation of cost and benefit (2) Guided implementation and re-evaluation of specific behavioral measures against challenging behavior. (3) Psychosocial support of MPS-families, including options for strengthening parental well-being and family functioning.

*Trial registration* This study was registered at clinicaltrials.gov prior to study start (NCT-Number: NCT03161171, Date: 2017/05/19).

**Electronic supplementary material:**

The online version of this article (doi:10.1186/s13023-020-01548-9) contains supplementary material, which is available to authorized users.

## Background

Mucopolysaccharidoses (MPS) are rare disorders. They represent the largest group of lysosomal storage diseases and are defined by defects in enzymatic degradation of glycosaminoglycanes [1, 2]. Depending on the affected enzyme, accumulation of particular metabolites causes typical organ and tissue impairment which is characteristic for the 7 MPS-types [2]. Neuropathology is common for MPS I, -II, and -III and -VII [2], of which the latter is not examined in this study due to its extreme rarity [1, 3].

Severity of affection is highly variable, especially for MPS I and -II, and must be considered as a continuum. Severe ends of this continuum are historically referred to as *Hurler syndrome* in MPS I [2, 4], and *severe Hunter syndrome* in MPS II [5, 6]. In both, MPS I and -II, neurodegeneration is regarded as distinguishing mark for severe disease presentation [4, 6]. MPS II almost exclusively affects boys due to x-linked recessive inheritance [2]. MPS III (*Sanfilippo syndrome*) is subdivided in subtypes-A to -D based on biochemical characteristics, with subtype-A being most common, showing earlier onset and faster progression of symptoms [2, 7, 8]. Severe neurodegenerative progression in MPS leads to significant reduction of life span [2, 7, 9, 10].

Severe MPS I and -II present early progressive somatic features such as skeletal deformities, coarse facial features, hernia, hepatosplenomegaly, and cardiac disease [2, 4, 6, 11]. MPS III, which is the most common MPS-type, shows only mild somatic involvement which often delays diagnosis [1, 2, 7, 8, 12]. Behavioral problems are frequent in MPS II and represent the major symptom in MPS III, within it is considered the most challenging aspect of the disorder aside from its devastating prognosis [13–15]. This challenging behavior includes sleep disturbance, hyperactivity, agitation, aggression, repeated behavior, unusual affect and apathy [13, 16]. Sleep disturbance is considered the most stressful and hardest to manage symptom and was associated with altered melatonin excretion (esp. MPS II, -III) and airway obstruction (esp. MPS I, -II) [2, 13, 14, 17–21]. Hyperactivity presents high parent wearing, with the maintenance of good physical strength in MPS III additionally hindering its manageability [12, 13, 15].

Enzyme replacement therapy (ERT] can have major impact on the course of somatic symptoms in MPS I and -II [22, 23]. However, as administered enzymes do not cross the Blood-Brain-Barrier (BBB), ERT is not suitable to prevent primary brain damage in MPS [24]. This problem is bypassed by intrathecal admission of enzymes (IT-ERT). Animal studies showed IT-ERT to be promising and implementation in MPS I, -II and -III patients is under evaluation [25]. To date, neuronal ceroid lipofuscinosis type 2 (CLN2) disease represents the only lysosomal storage disorder with successful implementation of IT-ERT [26]. Hematopoietic stem cell transplantation is being used in MPS I but requires early diagnosis and treatment [25, 27]. Future therapies may rely on in vivo genome editing which uses viral vectors to transmit of functional genes [28]. Animal studies have shown a good safety profile and promising effects on somatic and also behavioral symptoms. A key feature might lie in a long term high level enzyme titer, which may allow for a small but essential enzyme crossover at the BBB [29–31]. Individual immunologic response may limit the benefit of this approach [32]. Ongoing Phase I clinical studies address the implementation of in vivo-genome editing in humans [30, 31].

To date, the wide spectrum of symptoms in MPS demands a multi-professional treatment [5]. Physical factors may contribute to child behavior and must be clarified [15].

The devastating impact of disorder and especially of associated challenging behavior on affected families is known [13, 33, 34]. Whilst affected parents rate practical advice equally important as medical advice and behavioral approaches might be beneficial in the day-to-day management of challenging behavior, information about the effectiveness of specific procedures is lacking [13, 16, 35]. With the aim of offering affected parents specific effective day-today coping measures, this pilot study openly collected parental experiences in a cross-sectional, questionnaire-based regime. Therefore efforts were directed in investigating the following research questions:

### Research question 1

What is challenging behavior in MPS?

### Research question 2

Which strategies are helpful in the day-to-day coping with challenging behavior?

### Research question 3

How strong is parental acceptance of illness and the disorder’s impact on family relationships?

### Research question 4

What are beneficial personal and interfamilial strategies for generally coping with the disorder?

## Methods

This explorative, cross-sectional study was approved by the ethics committee of the medical faculty of the University of Heidelberg. STROBE-criteria were respected (Strengthening the Reporting of Observational Studies in Epidemiology; http://www.strobe-statement.org).

In cooperation with the German MPS Society, a convenience sample of all registered families of children with MPS I, -II and -III and neurological involvement (n = 268) were invited to participate by mail in the summer of 2017. Written informed consent was obtained. Thirty-seven questionnaires were returned (rate: 13.8%). Of these, 34 (MPS I: n = 8 (MPS IH: n = 7; MPS IS: n = 1), MPS II: n = 8; (severe subtype: n = 5; subtype unknown: n = 2; mild subtype n = 1) MPS III: n = 18 (MPS IIIA: n = 11; MPS IIIB: n = 7)) met the inclusion criteria: Reported diagnosis of MPS I, -II or -III, reported developmental delay or neurodegeneration, age < 18 and written informed consent (flowchart in Additional file 1). Neurological involvement is a hallmark of severe disease in MPS I and -II [4, 6]. The questionnaire prompted developmental delay *(“does your child lag in skills compared to healthy peers?”*) and neurodegeneration/developmental regression (*“has your child lost skills it previously had?*”) By preselection of neurologically affected children according to our inclusion criteria, this study created a focus on severe cases in these subtypes. Two questionnaires of MPS-II children stated an unknown subtype, one questionnaire of MPS-I reported a *Scheie* subtype. As developmental affection was reported in these individuals, they were included in the study. Only one child had a history of MPS-specific therapy.

De novo design of the study questionnaire included input from affected families. The questionnaire had to meet the fine line between an open and unbiased assessment of parental experiences on one hand, and the collection of comparable qualitative data on the other. The questionnaire pre-review by affected families showed semi-structured items with the use of both direct and open requests for symptoms as well as an open assessment of coping strategies supported by exemplary strategies to be the most conducive in achieving this goal.

Next to illness-related and socio-demographic data, the questionnaire collected relevant challenging behavior by frequencies and respective subjective parent- and child stress. Literature and personal exchange with affected parents highlighted sleep disturbance, aggression, hyperactivity, agitation, repeated behavior, unusual affect and apathy as common symptoms in neuropathological MPS [13, 16]. To ensure consistency and comparability of data, these symptoms were directly prompted. Participants were invited to additionally report and rate further relevant symptoms.

Practical coping strategies against challenging behavior were openly surveyed and included ratings of their perceived effectiveness. To support participants in this task, boxes of exemplary strategies, as defined by the authors and affected families, were provided. To allow for comparison, the questionnaire asked for strategies against sleep disturbance, hyperactivity, aggression and repeated behavior whilst providing further pages to collect strategies against additional individual symptoms.

To evaluate the disorders’ influence on personal and interfamilial wellbeing, participants were asked to rate their personal acceptance of illness and the impact of the disorder on family relationships. Assessment of strategies for generally coping with the disorder included their perceived effectiveness. In collaboration with affected families, promising strategies for personal coping (*sports, distraction/time*-*out outside the family, homeopathy, psychotherapy)* and interfamilial coping (*joint excursions, mutual support with child care, homeopathy, psychotherapy)* were defined. Assessment of these strategies was complemented by blank spaces inviting ad hoc individual strategies. This approach provided an incentive for open comments and at the same time allowed for comparisons of likely functional strategies.

Alongside with free text investigations, this approach supported a wide collection of parental experiences in the sense of an explorative study. Ratings used Visual Analogue Scales (VAS; Range 0.00–5.00) and were interpreted as shown in Table 1.

**Table 1:** Interpretation of reported VAS-values

	Research Question 1 and 2			Research Question 3	Research Question 4
	Symptom frequency and regarding stress			Coping strategies	Acceptance of illness
Range	Frequency	Parent stress	Child stress	Effectiveness	Acceptance
0.00–1.66	Mild			Low	
1.67–3.33	Moderate			Intermediate	
3.34–5.00	Severe			High	

Symptom frequencies refer to the study sample, whereas reported parent- and child stress levels relate to the subgroup of individuals who reported the presence of the symptom at least with moderate symptom frequency. Correlation and comparison testing included all individuals. Direct comparison of directly prompted and openly reported symptoms must be conditional, as illustrated in the discussion. Impact on interfamilial relationships used two-sided VAS-scales (range −2.50 to +2.50). Advice for ‘recently diagnosed’ families was collected through free text investigation.

The open character of the study generated a wide quantity and variety in reported coping strategies. For operationalization, reported practical coping strategies have subsequently been categorized with the help of 3 affected mothers as follows:

- “Distraction/Busying”: measures to distract the child or keep him/her busy.
- “Relief/Safety”: measures fostering child relief and feeling of safety.
- “Frame Conditions”: measures to adapt situational frame conditions.
- “Operant Conditioning“: measures aiming at changing behavior through reinforcement and punishment.
- “Professional Therapy”: therapeutic interventions, such as equine-assisted therapy or music therapy.
- “Breathing Support”: measures to facilitate child breathing (sleep disturbance only).
- “Medication”: medicamentous approaches to treatment.

To enable comparison of medication- and non-medication-approaches, all measures that could not be attributed to “Medication” were pooled in the category “Non-medication”.

Five subgroup analyses of practical coping strategies were conducted for the following seminal issues: (1) sleep disturbance (n = 29), (2) hyperactivity (n = 23), (3) aggression (n = 16), 4) repeated behavior (n = 15), 5) orality (n = 5). In these analyses, the denominator for the shown frequencies refers to the proportion of individuals who provided data for coping strategies related to the above seminal issues. Of those, we considered coping approaches relevant, if they were either (a) reported by ≥ 50% or (b) reported by ≥ 33% and, at the same time, were rated at a mean efficacy of ≥ 3.34 on the VAS scale. Single measures are not listed. All reported measures and corresponding categorization are presented in Additional file 2.

Comparison of measures in the form of financial and temporal cost/benefit analyses was initially planned but revealed to be inconclusive due to huge variability of data. Owing to the small sample size, data is displayed covering all MPS-types pooled together, subsequently indicating peculiarities between types if existent. To meet the progressive character of the disorders, an Ability Score was created to approximately display child abilities as the sum of three items: best possible mobility, -language and -feeding (range 0 (low abilities) to 6 (high abilities), also see Additional file 3).

Given the rarity of the disorders and the questionnaire design which included open-ended questions, techniques of descriptive statistics were applied. With the use of SPSS V.24, inferential analysis contained distribution location comparison (Kruskall-Wallis/Mann–Whitney-U) and correlation analysis (Spearman), applying a confidence interval of 95%. Available case analysis was used to handle missing data. Alterations in sample size are indicated at appropriate points. Testing for outliers utilized scatterplots. Due to the exploratory nature of the investigation and the limited sample size, no adjustment for multiple comparison was conducted and results are reported as ’significant’ at the unadjusted level of 5% [36–38].

## Results

Questionnaires were completed by mother (n = 28), both parents (n = 3), father, grandmother or sister (n = 1 each). Reported children were male in 52.9% (n = 18) with mean = 8.7 (SD = 4.23) years of age.

Analyzed questionnaires (n = 34) reflect the frequency distribution of neuropathological MPS-types in Germany (MPS I, -II: 23.5% each; MPS III: 52.9%) [1]. For further information see Table 2.

**Table 2:** Child- and illness-related data

	MPS I (n = 8)		MPS II (n = 8)		MPS III (n = 18)		Overall (n = 34)	
	mean n	SD (%)	mean n	SD (%)	mean n	SD (%)	mean n	SD (%)
Age	8.2	4.0	6.8	4.5	9.8	4.1	8.7	4.2
*Gender*								
f	6	(75.0)	0	(0.0)	10	(55.6)	16	(47.1)
m	2	(25.0)	8	(100)	8	(44.4)	18	(52.9)
Age at first symptoms (yrs)	0.5	0.4	0.9	0.6	2.0	2.1	1.4	1.7
Age at diagnosis (yrs)	1.8	2.0	2.2	0.9	4.5	2.4	3.3	2.3
Developmental delay	8	(100)	8	(100)	18	(100)	34	(100)
Developmental regression	4	(50.0)	4	(50.0)	15	(83.3)	23	(67.7)
Ability Score	5.4	0.5	4.5	2.1	3.4	1.9	4.2	1.9
Mobility	1.5	0.5	1.6	0.7	1.2	0.9	1.4	0.8
Speech	1.9	0.4	1.3	0.9	0.7	0.8	1.1	0.9
Feeding	2.0	0.0	1.6	0.7	1.6	0.6	1.7	0.6

### Research question 1: Challenging behavior: Frequency, parent- and child stress

**Fig. 1:**
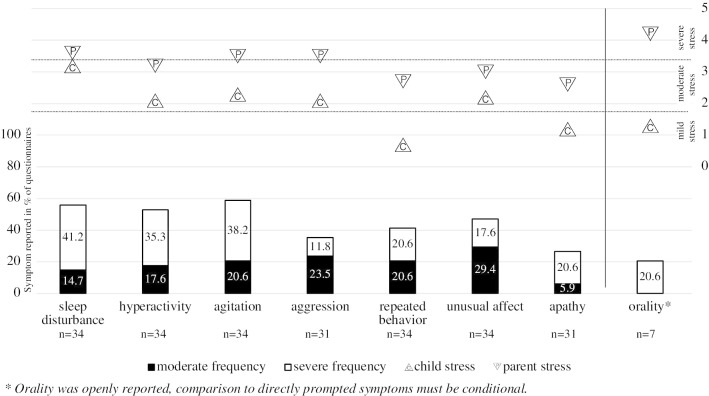
MPS I-III: symptom frequency; respective stress if symptom is present. Symptom frequency in  % of all questionnaires (n = 34); respective stress levels if symptom is existent (at least with moderate frequency) as reported on VAS with range 0.00 (low) to 5.00 (high)

The relevance of sleep disturbance, hyperactivity and agitation showed in their reported frequency and respective parent stress (as well as child stress for sleep disturbance). Additionally, aggression seemed relevant given severe parents stress despite low frequency. Further, orality (i.e. child putting things in its mouth) appeared to be relevant due to multiple indication despite open questioning. Repetitive behavior, unusual affect and apathy appeared less relevant against the background of mild to moderate frequency and respective parent- and child stress (Fig. 1).

The differences between perceived parent/child stress where significantly different from 0 (one-sample Wilcoxon signed rank test on the differences) for all reported symptoms except for sleep disturbance.

Overall, sleep disturbance was rated with moderate symptom frequency (n = 34; mean = 2.2). Reported parent- and child stress were rated highest in all directly prompted symptoms. Hyperactivity was rated with moderate symptom frequency (n = 34; mean = 2.1). Respective parent- and child stress were evaluated as moderate. Agitation was rated with the highest frequency in all directly prompted symptoms (n = 34; mean = 2.4) and was associated with severe parent stress and moderate child stress. Despite its overall reported mild frequency (n = 31; mean = 1.2), aggressive behavior was rated with severe parent stress and moderate child stress. Even though orality/preservative chewing was not explicitly questioned, it was reported in 7/34 individuals (20.6%) and rated severe in frequency and parent stress and mild in child stress. Repeated behavior was absent in MPS I, leading to an average mild reported frequency (n = 34; mean = 1.6). Respective parent stress was moderate, child stress was evaluated mild. Repeated behavior was described in 14 questionnaires as repetitively activating light switches, opening/closing doors, windows, shutters, refrigerators or toilet lids as well as repetitively throwing objects, activating water taps or flushing toilets. Unusual affect was overall rated as moderately frequent (n = 34; mean = 1.7) with moderate parent- and child stress. The symptom was described in three questionnaires as whining or shouting. Apathy was rated with mild frequency (n = 31; mean = 1.3) and child stress, respective parent stress was rated moderate. Reported apathy did not correlate with child age (rs = .11; p = .951) or Ability Score (rs = − .26; p = .161).

Average reported symptom frequency was rated lower in MPS I. Differences were significant for MPS I versus MPS II in hyperactivity (U = 6.0; p = .004), repeated behavior (U = 11.5; p = .023) and agitation (U = 9.0; p = .014) as well as for MPS I versus MPS III in repeated behavior (U = 21.0; p = .002) and agitation (U = 16.0; p = .001). In MPS I, sleep disturbance and aggression were the only symptoms repeatedly reported in relevant frequency (n = 3 each). Respective parent- and child stress were rated moderate. MPS II scored highest in all MPS-types in frequency of hyperactivity, agitation, aggression (n = 7; mean = 1.7), repeated behavior (n = 8; mean = 2.1), unusual affect (n = 8; mean = 2.1) and orality (n = 3; mean = 5.0). MPS III scored highest in all MPS-types in frequency of sleep disturbance and apathy (n = 17; mean = 1.6). Parent stress in MPS III was rated severe for sleep disturbance, hyperactivity, agitation, aggression (n = 5; mean = 3.4), apathy (n = 6; mean = 3.7) and orality (n = 4; mean = 4.5). Table 3 presents highest reported frequencies and parent-/child stress of directly prompted symptoms for each MPS-type (also see Additional file 4). Differences between subtypes of MPS II and -III were insignificant in all symptoms with one exception: In MPS IIIB parent stress (mean = 2.3 vs. mean = 4.4; H = 5.83, p = .014) and child stress (mean = 1.1 vs. mean = 3.0; H = 5.89, p = .013) due to agitation were rated significantly higher in with insignificant difference in respective symptom frequency (mean = 3.1 vs. mean = 2.7; H = 0.13, p = .740). The Scheie patient showed mild symptom frequencies and respective stress in parents and the child. One individual had received MPS-specific therapy prior to this study. Reported symptom frequencies and respective stress levels this child and its parents were low in all symptoms.

**Table 3:** Highest reported symptom frequency, parent stress and child stress

MPS I				MPS II			MPS III			Overall		
Symptom		n	Mean	Symptom	n	Mean	Symptom	n	Mean	Symptom	n	Mean
*Highest reported symptom frequency*												
Sleep disturbance		8	1.4	Hyperactivity	8	3.5	Agitation	18	2.9	Agitation	34	2.4
Aggression		8	1.0	Agitation	8	3.5	Sleep disturbance	18	2.5	Sleep disturbance	34	2.2
Unusual affect		8	0.7	Sleep disturbance	8	2.3	Hyperactivity	18	2.2	Hyperactivity	34	2.1
*Highest reported parent stress*												
Aggression		3	3.0	Aggression	4	3.9	Hyperactivity	10	4.0	Sleep disturbance	19	3.6
Sleep disturbance		3	2.6	Sleep disturbance	5	3.8	Sleep disturbance	11	3.8	Agitation	20	3.5
				Agitation	6	3.2	Agitation	13	3.7	Hyperactivity	18	3.5
*Highest reported child stress*												
Sleep disturbance		3	3.2	Sleep disturbance	5	2.6	Sleep disturbance	11	3.5	Sleep disturbance	19	3.2
Aggression		2	3.2	Agitation	6	2.0	Hyperactivity	10	2.8	Agitation	20	2.3
				Hyperactivity	7	1.5	Unusual affect	11	2.5	Unusual affect	16	2.2

Frequency in all individuals; mean parent stress and child stress if symptom is existent (at least with moderate frequency); mean values as reported in VAS (range 0.0–5.0). Single ratings are not listed

Orality not included in this table as direct comparison may be misleading (see discussion)

### Research question 2: Day-to-day practical coping with challenging behavior: measures and effectiveness

Practical coping strategies for sleeping disorders, hyperactivity, aggression, and repeated behavior were numerous. Despite open questioning, measures against orality were repeatedly reported. Due to little data concerning agitation, unusual affect, apathy and other symptoms, reported coping strategies are not further illustrated.

Eighty-five percent of all questionnaires reported measures against sleeping disorders with an overall moderate perceived effectiveness (n = 29; mean = 3.3). Most coping strategies related to the adaption of “Frame Conditions” (n = 24/29; mean = 3.2). Frequent individual measures in this category were *day*-*time exercise* (n = 13/29; mean = 2.7) and *changing type of bed* (n = 12/29; mean = 3.7), of which the latter ranked most effective in all measures. Strategies aiming at “Relief/Safety” were described in n = 17/29 individuals and rated with intermediate effect (mean = 3.0). *Letting child sleep in parent bed* (n = 11/29; mean = 2.7) was the most common individual measure in this category. “Medication” (n = 14/29; mean = 3.4) was rated highly effective in the treatment of sleep disturbance with *melatonin* being the most frequent but least effective individual measure (n = 8/29; mean = 2.1). Measures in “Breathing Support” (such as *Continuous positive airway pressure (CPAP), oxygen* and *inhalation therapy*) were reported in individuals with MPS I only and were rated highly effective (n = 2/29; mean = 4.7).

Measures against hyperactivity were given in 68% of all questionnaires (n = 23) and were overall rated highly effective (mean = 3.4). “Distraction/Busying” (n = 22/23; mean = 3.3) was rated moderately effective and contained individual measures such as *exercise* (n = 16/23; mean = 3.3) and *singing* (n = 8/23; mean = 3.4). The adaption of “Frame Conditions” (n = 14/23; mean = 3.4) was rated highly effective with *regular daytime routine* (n = 14/23; mean = 3.4) being the most common individual measure. “Relief/Safety” (n = 10/23; mean = 3.9) was the category with the highest rated effect, including the use of a *seat belt* (n = 6/23; mean = 4.5) as the most frequent individual measure.

Measures against aggression were reported in 47.1% of all questionnaires (n = 16) and were rated with the least overall effect in all symptoms (mean = 3.0). Three categories were equally frequent in use (n = 12/16): “Distraction/Busying” (mean = 3.4) containing *exercise* (n = 6/16; mean = 2.6), “Relief/Safety” (mean = 3.0) with *calming child down* (n = 9/16; mean = 2.7) as well as “Operant Conditioning” (mean = 2.4) including *rejection of aggression* (n = 8/16; mean = 2.4).

44.1% of all questionnaires (n = 15) named measures against repeated behavior, with the overall effectiveness being highest in all symptoms (mean = 3.6). Adaptation of “Frame Conditions” scored high in terms of effectiveness (n = 11/15; mean = 4.0). Most individual measures in this category aimed at the avoidance of repeated behavior (n = 9/15), such as *locking doors* (n = 3/15; mean = 4.6) or *masking light switches* (n = 2/15; mean = 4.8). “Distraction/Busying” was rated intermediately effective (n = 10/15; mean = 3.2) with *keeping child busy* (n = 7/15; mean = 3.5) being the most frequent individual measure.

In 5 of the 7 questionnaires reporting orality, respective coping measures were found (mean = 3.1). “Distraction/Busying” was rated highly effective (n = 4/5; mean = 3.4) with the use of *teething rings* (n = 3/5; mean = 3.8) as the most frequent individual measure. Only few questionnaires contained measures against agitation (n = 2, 5.9%) or unusual affect (n = 4, 11.8%) whilst measures against apathy were not reported. Some questionnaires named measures against further symptoms (also see Additional file 2). Comparison of MPS-types showed the overall effectiveness of measures to be high in all symptoms in MPS I, intermediate to high in MPS II and intermediate in all symptoms in MPS III.

The use of “Medication” as a coping strategy was reported in 47.1% of questionnaires (n = 16) and predominantly related to the therapy of sleeping disorders and hyperactivity. In the treatment of sleeping disorders, “Medication” (n = 14/29; mean = 3.4) scored slightly higher in perceived effectiveness than “Non-Medication” (n = 28/29; mean = 3.2). *Melatonin* was reported the most frequent but least effective single drug (n = 8/29, mean = 2.1). All reported antipsychotics were rated highly effective (n = 8/29; mean = 4.5). With respect to hyperactivity, “Medication” (3/23; mean = 3.4) and “Non-Medication” (23/23; mean = 3.4) were equally rated highly effective (also see Additional file 5).

### Research question 3: Acceptance of illness and impact of the disorder on family relations

Overall, participants reported an intermediate acceptance of illness (n = 33; mean = 2.7) with insignificant differences between the MPS-types (H = 16.1; p = .872). Reported acceptance significantly correlated with child age (rs = .51; p = .003) and time span since diagnosis (rs = .55; p < .001). Correlation with average frequency of reported symptoms (rs = −.10; p = .575), associated overall parent- (rs = −.08; p = .653) or child stress (rs = −.05; p = .767) was not significant. Regarding the Ability Score, significant negative correlation with reported acceptance was found in MPS III (rs = −.65; p = .003). When analyzing the whole sample, this significance was given for the sub-item of mobility only (rs = −.40; p = .020). Correlation with manageability of the financial burden due to MPS (rs = .10; p = .571) and differences in acceptance depending on parental relation status (U = 83.0; p = .738) were insignificant. Impact of the disorder on the relationships with partner (n = 31; mean = 0.2), healthy siblings (n = 20; mean = −0.4) and extended family (n = 32, mean = 0.2) was rated marginal with minimal differences within MPS-types (also see Additional file 6).

### Research question 4: Personal and interfamilial coping with the disorder: measures and effectiveness

Among strategies reported for coping with the child’s disorder, *communicating with friends, relatives, and acquaintances* (n = 26; mean = 3.4) ranked highest in effectiveness. *Time*-*out alone* was rated highly important (n = 27; mean = 4.5) with *Distraction/time*-*out outside the family* being rated intermediately effective in coping with the disorder (n = 28; mean = 3.3). *Psychotherapy* (n = 14; mean = 1.5) and *homeopathy* (n = 8; mean = 0.0) were rated low in effectiveness. Despite open questioning, *communication with MPS*-*families* (n = 4; mean = 4.2), *information about MPS* (n = 3; mean = 4.1) and *hospice stays* (n = 3; mean = 4.5) were reported repeatedly and rated highly effective.

Regarding strategies for strengthening family relationships, *mutual support with child care* (n = 24; mean = 3.9) and *joint excursions* (n = 20; mean = 4.0) were reported most frequently and rated highly effective. *Homeopathy* (n = 3; mean = 0.0) was rated low in effectiveness whilst *psychotherapy* (n = 7; mean = 2.1) was rated intermediate. Within openly collected measures, the *creation of free space in partnership* (n = 8; mean = 4.0), *time out alone* (n = 3; mean = 4.3) and *open communication* (n = 3; mean = 4.5) were rated highly effective.

Free text investigation concerning advice for ‘recently diagnosed’ families was completed by 31/33 families. Advice mostly related to *networking with other affected families* (n = 17), *acceptance of illness* as well as *contacting specialists/activating resources in one‘s surroundings* (n = 13 each). Measures further included advice for *communicating/strengthening family relationships* (n = 6) *interaction with the child* (n = 7) and *seeking psychotherapeutic/psychiatric help* (n = 5). Reported coping measures and advice are presented in Additional file 6).

## Discussion

Challenging behavior is known as a core symptom in neuropathological MPS and puts a major strain on affected families [7, 13, 21, 33, 39]. Whilst behavioral coping strategies might be beneficial in the treatment of challenging behavior in MPS there is only little related data published [35]. Further investigation regarding their effectiveness is needed [16]. This study collected day-to-day practical coping strategies for challenging behavior as well as strategies for coping with the burden of the disorder, with the aim of offering affected families specific possible solutions in these complex situations.

### Research questions 1 and 2: What is challenging behavior in MPS and which strategies are helpful in the day-to-day coping with it?

Relevance of symptoms was deducted by symptom frequency, parent stress and child stress [40]. Sleep disturbance, hyperactivity, and agitation seemed relevant by ratings of frequency and parent stress [as well as child stress in sleep disturbance). In addition, aggression and seemed significant by severe parent stress. Despite open questioning, orality was indicated repeatedly and rated severe in frequency and parent stress. Practical coping strategies were overall rated intermediately to highly effective with measures against repeated behavior being most effective in all symptoms.

Results show distinct differences in symptom frequency in-between MPS-types. MPS I clearly showed lower symptom frequency and respective parent- and child stress alongside higher efficiency of reported measures. This finding corroborates previous reports which attributed a rather gentle and calm temperament with children affected by MPS I [2]. Discrepancy of reported effectiveness of measures in between MPS-types might result from variation in severity of symptoms, but also from unequal implementation or conception of measures [35].

Subtypes of MPS II and -III showed only insignificant differences regarding symptom frequency and respective stress. In agitation only, ratings of parent- and child stress in MPS-IIIB significantly exceeded respective values in MPS IIIA. This might be interpreted is indicating a larger burden caused by agitation in MPS IIIB. However this may rather be a random finding, as there was only a slight difference in reported frequency of agitation and previous studies indicate a more severe course of disease in MPS IIIA [7].

The Scheie patient and the previously treated patient in our study showed mild symptom frequencies and respective stress in parents and children. This might be interpreted as less severe affection and good effect of the received therapies respectively, however a subgroup of n = 1 does not allow for robust comparisons.

### Sleep disturbance

Sleep disturbance is known as a core symptom in MPS I, -II and -III and often shows high parent-wearing [13, 14, 20, 35, 41]. Despite its partly lower reported frequency compared to foregoing studies, sleep disturbance has been rated severest in all directly prompted symptoms regarding parent- and child stress. [11, 13, 14, 21]. Sleeping problems appear to be one of the most difficult symptoms in this disorder, as previously reported [14, 19].

This may also reflect reported practical coping strategies as the most numerous in all symptoms. Results confirm previous research stating the overall effect of strategies against sleep disturbance to be acceptable to very acceptable [20]. Most reported strategies aimed at adaption of frame conditions and the support of the child’s relief and feeling of safety. These strategies have overall been rated intermediately effective, with changing the type of bed as the most effective individual measure. Others included daytime-exercise and child sleeping in parent bed. The benefit of the latter was questioned by Fraser et al., as sleeping separately is seen to improve parent sleep and consecutive ability to manage the disorder the next day [20]. Sleep disturbance appears secondary to sleep apnea in MPS I and primary CNS disease in MPS III respectively. In MPS II, both mechanisms seem to play a role [14, 17, 42, 43]. Strategies of breathing enhancement (CPAP, oxygen and inhalation therapy) were reported in individuals with MPS I only and seemed highly effective. Medication therapy was rated highly effective in the treatment of sleeping disorders. Antipsychotic medication was frequently reported and ascribed high effectiveness. However, its use must be considered against the high risk of side effects such as over-sedation or hangover the next day [8, 15, 21, 35]. Although melatonin was the most frequently reported single drug in this study, it was rated least effective in all medicaments. Previous studies have accentuated the substitution of melatonin in the treatment of sleep disturbance, as it interacts with the impaired circadian melatonin rhythm in MPS II and -III and is considered comparably safe in use [18, 20, 44]. Medication and non-medication approaches to treatment may be considered equivalent pillars in the treatment of sleep disturbance, given their similar ratings of effectiveness. The lack of possible side effects further encourages tapping the full potential of non-medication approaches.

### Hyperactivity

In line with previous studies, hyperactivity was reported rare in MPS I but seemed to be of high relevance in MPS II and -III [2, 12, 13, 21, 45]. In MPS II, hyperactivity seemed relevant by high frequency, whereas in MPS III its relevance became clear by severe ratings of parent stress. The highly parent-wearing character of hyperactive behavior in MPS III has been reported previously and may be a result of long preservation of motor skills in this MPS-Type [12, 13, 15]. Contradictorily, mobility was reported higher in MPS II. Factors mediating hyperactivity and respective parent stress need further investigation. The stage of illness must be considered when analyzing hyperactivity in MPS.

Day-to-day coping strategies against hyperactivity have overall been rated highly effective. Measures aiming at distraction, busying and relief have (next to medication) been reported in previous literature but effectiveness has only been rated for overall measures [13]. This study examined practical strategies in detail and could highlight the adaption of frame conditions as an additional effective strategy. Individual measures such as frequent daytime-exercise, daily routine, singing and busying the child seem worthwhile when dealing with hyperactivity in MPS. Non-medication approaches to the treatment of hyperactivity were overall reported as predominant in use and rated highly effective. This contrasts with previous findings which claimed a low response to behavioral approaches in MPS III [8]. This study may put behavioral measures into perspective, not least considering possible side effects of medicaments such as paradox overactivity [8].

### Agitation

Reported frequency and parent stress underline the high relevance of agitation in MPS. However, only few coping strategies against this symptom have been reported by parents. This may be a result of strategies which were not directly questioned. Furthermore, there might be an overlap to hyperactivity in both, applied measures and semantic aspects (given the chosen German translation “Unruhe” for agitation). As agitation has been prompted after hyperactivity, this might have prevented parents from stating further measures. Future investigation should offer exact definitions to eliminate possible interferences.

### Aggression

The relevance of aggression becomes evident in parent stress being reported severe and scoring highest in all directly prompted symptoms regarding MPS I and -II, despite its overall ratings of mild frequency. Parents being recipients of the aggression may play a role in this remarkable result. However, as Malcolm et al. have reported, parent–child-interaction concerning aggression might be more complex. With knowledge of their child’s restricted communication skills and lacking awareness of negative aspects of aggressive behavior, parents need to oppress their own emerging emotions such as anger or sadness. This may result in a feeling of guilt, when parental tolerance is exceeded [13]. The very complexity in this interaction might intensify parent stress. Stigmatization of aggression in the society might have accounted for low reported frequency and above average omission of items.

Given the impaired cognition of the child, the understanding of aggression as symptomatic might be of fundamental importance when coping with this symptom [13]. This may also show in reported effectiveness of practical coping strategies: Whilst strategies involving distraction and busying as well as fostering the child’s relief and feeling of safety seemed most effective, responsiveness to operant conditioning was rated intermediate. Practical manageability of aggression seems restricted, as it was hindmost in all symptoms regarding effectiveness of reported measures. This may not least be attributed to the high ratings of parent stress. Further investigation is needed to improve parental support in dealing with this stressful symptom.

### Orality

Orality is a common symptom in MPS and is partly referred to as low-level repetitive behavior [42, 46, 47]. In this study it stood out due to frequent indication despite open data collection. Results might be biased by its use as showcase in questionnaire instructions (priming-effect). Orality was reported in MPS II and -III only and rated severe in frequency and parent stress. These average ratings were highest in all symptoms, however, direct comparison to other symptoms must be conditional: As the questionnaire prompted “further relevant symptoms”, open report may have selected severe cases, so frequency and parent-stress may be overestimated and should be evaluated in future research. Overall effectiveness of coping strategies was rated intermediate with the use of biting rings as the most frequent and effective measure.

### Repeated behavior

Parent stress due to repeated behavior was rated moderate, which may be due to the effectiveness of reported coping strategies being highest in all symptoms. The most frequent and effective reported individual measure was busying the child. Environmental change was reported the most effective category and included measures taken to prevent repeated behavior (such as masking switches or locking doors).

In this study, repeated behavior was reported in more than 50% of individuals with MPS II and -III, even though orality was analyzed separately. Repetitive behavior has been described in previous case reports of MPS and in the authors personal communication with affected families [48, 49]. However, foregoing investigation could not detect repetitive behavior using ADOS (Autism-Diagnostic-Oberservation-Schedule) and therefore it is not considered symptomatic for MPS [50, 51]. There seems to be a discrepancy in parental perception of repeated/repetitive behavior versus its definition within autism spectrum-disorders. As mentioned above, parents seem to have developed effective strategies for the management of their child’s repeated behavior. To allow distribution and exchange of respective parental experiences, it may be necessary to consider the behavior as symptomatic for the disorder, however it may be referred to. Further investigation into repetitive/repeated behavior in MPS II and -III is needed for this step.

### Other symptoms

Unusual affect and apathy seemed less relevant due to reported frequency and parent stress. Respective practical coping strategies were hardly ever reported. Although ongoing cognitive and moto-neural decline often lead to a vegetative state in the later phase of illness for MPS II and -III, reported apathy did not show a significant decline with child age or loss of abilities [2, 7, 52]. This may be a result of a comparatively good state of health in this study sample, according to the Ability Score. Parents reported further behavioral and non-behavioral symptoms (see Additional file 2).

### Research questions 3 and 4: How strong is parental acceptance of illness and the disorder’s impact on family relations? What are beneficial personal and interfamilial strategies for generally coping with the disorder?

Acceptance of the child’s illness was rated intermediate and did neither seem to be generally affected by socio-demographic aspects such as financial burden incurred by the disorder or actual parental relation status, nor by illness-related factors such as MPS-type, frequency of challenging behavior or respective parent- or child stress. Acceptance even partly increased with decreasing child abilities. It rather seemed to be correlating with the child’s age and time span since diagnosis. These findings may implicate that with time, parents will grow into the acceptance of having a child affected by MPS and that this development is largely unaffected by child behavior or state of illness. Consciousness of this might be of value regarding personal coping abilities of affected parents. The importance of acceptance of illness for parental wellbeing has been displayed in previous studies and reflects in the free text investigation of advice for ‘recently diagnosed’ families, which found the development of acceptance to be the second-most frequent suggestion given [34, 53].

Social exchange and private space seemed highly relevant when coping with MPS. The importance of social resources as a protective factor has been highlighted before [34]. Accordingly, communication with friends and relatives has been rated the most effective personal coping strategy. Networking with other MPS-affected families offers room for practical and social exchange. It was the most frequent advice given and rated very important. Next to social exchange, private space such as time-out has been reported highly important and effective in coping with the disorder.

Private space also seemed to be of significance for strengthening interfamilial relations. Time-out alone as well as free space for partnership were frequently reported and rated highly effective. Raina et al. [54] highlighted the importance of family functioning when caring for a child with mental disorders. To support interfamilial relationships, mutual support with child care, joint excursions and open communication seemed highly effective measures. It may be due to the effectiveness of parental coping strategies that the influence of the disorder on inner and extended family relations has been rated low in all MPS-types.

## Limitations

One limitation of this study consists in the small sample size which affected statistical analyzability and is determined by the rarity of the disorder as well as the required registration at the German MPS Society. The novel research question and explorative character of the study required an open questionnaire, which needed to be designed de novo. The authors consider the involvement of affected parents in the questionnaire design a particular strength of this study [55]. The explorative character of this investigation initially intended an entirely open questionnaire. The pre-review by affected parents, however, resulted in the implementation of a semi-structured questionnaire, with the use of showcases and partly closed questioning. This approach might have enhanced respective indications, however, it showed to be more conducive to achieving the ambition of the study. The study focused on the explorative assessment of subjective parental experiences. Externalizability should be confirmed in future investigations. The questionnaire enabled a wide collection of parental experiences. However, in combination with the small sample size, the variety of data often resulted in small subgroups that would elude inferential statistics. Available case analysis was used to handle missing data. This approach assumes missing data to be completely random, which might have biased the results. Future research should use multiple imputation as reliable tool to handle missing data that is not missing completely at random [56–58]. Reliability of displayed statistics must be considered against the above portrayal. Reported coping measures were manifold and demanded subsequent categorization to allow interpretation. Its implementation was based on subjective valuation of measures by affected parents and the authors which might have influenced results. An important factor influencing the validity of the collected data is how well the questionnaire captured parental experiences. The questionnaire format allowed to target a wide range of potential participants with a relatively low logistical burden and offered stable survey conditions. An interview or observational format might have allowed for a more individual inquiry of parental experiences but would have required more resources. Limitations of sample size and required questionnaire design result in limited internal and external validity and demand confirmation in future research.

## In summary

Challenging behavior is known as a core symptom in neuropathological mucopolysaccharidoses alongside with its extensive effect on the well-being of affected families [7, 13, 21, 33, 39]. Literature highlights the urge for investigation into possible solutions in the management of challenging behavior in MPS [16, 35]. This study could provide a first step towards the collection of effective practical strategies for coping with challenging behavior in MPS. Given the explorative character of the study, alongside with its limitations, the validity of findings should be confirmed in case control studies to allow implementation in the every-day life of affected families.

In line with previous findings, challenging behavior in MPS II and -III seemed considerably more frequent and difficult to manage than in MPS I [2, 14, 16]. Sleep disturbance, hyperactivity and agitation are reported highly frequent and stressful symptoms. Furthermore, aggression and orality need to be considered relevant due to major parent stress.

Distraction, relief, the adaption of frame conditions and medication might be effective strategies when dealing with sleep disturbance and hyperactivity in MPS. Similar effectiveness ratings of medication- and non-medication-strategies highlight the importance of the latter, particularly against the background of absent side effects. Manageability of aggression seems limited which might be one reason for high respective parent stress. Worthwhile approaches might include distraction techniques and reassurance rather than operant conditioning. Parents frequently reported repeated behavior as part of their child’s actions although repetitive behavior is not considered symptomatic for MPS [59]. They seem to have developed effective strategies in the management of this behavior. Transmission of these strategies to inexperienced affected families might require to accredit the behavior as symptomatic for the disease, however it may be referred to.

Results are in line with previous findings, highlighting the importance of acceptance of illness for parental well-being [34, 53]. Parents seem to grow into this state of acceptance, largely uninfluenced by child behavior or state of illness. Awareness of this could encourage their personal coping abilities. Social exchange and private space seemed important to strengthen personal and interfamilial functioning. Networking with other affected families appears to be crucial when dealing with a rare disorder like MPS.

## Conclusions and perspective

Findings of this study implicate that parents could benefit from a multimodal mentoring plan including:

*First* day-to-day practical coping strategies against challenging behavior. A register of specific measures could help affected families to implement effective day-to-day coping. Diary documentation could help practitioners and families to evaluate strategies.

*Second* pharmacological treatment of challenging behavior such as sleep disturbance and hyperactivity, carefully considering possible side effects.

*Third* supervised support of familial resilience. Allowance of personal and interfamilial space and motivation towards social interconnection seem crucial in this regard. In this context, networking with other affected families is highly recommendable.

Assessed strategies may further be of use for the adaption of existing parenting programs for challenging behavior in children with intellectual disabilities to the special needs in MPS [15, 60].

This study could provide a first step towards the exploration of possible strategies for coping with challenging behavior in MPS and the complex burden that derives from the disorder. Future investigation is needed to confirm findings and allow implementation in families everyday lifes.

## Supplementary information

**Additional file 1**: Flowchart. Description: flowchart on how the sample size was arrived.

**Additional file 2**: Reported practical coping measures–categorization and perceived effectiveness. Description: Tabular presentation of reported practical coping measures against challenging behavior (2a-i), their perceived effectiveness and subsequent categorization.

**Additional file 3**: Ability Score–calculation and results. Tabular presentation of calculation of the Ability Score (3a) and results in individual MPS-types (3b).

**Additional file 4**: Reported symptoms–frequency, parent- and child stress. Presentation of reported symptoms in individual MPS-types in 3 tables: frequency (4a), parent stress (4b) and child stress (4c).

**Additional file 5**: Reported medication therapy. Tabular presentation of reported Drug therapy against challenging behavior and its perceived effectiveness, as well as comparison of total effectiveness of medication- and non-medication-measures.

**Additional file 6**: Coping the disorder. Description: Tabular presentation of reported acceptance of illness and personal coping strategies (6a), the disorders impact on family relationships and strategies for strengthening them (6b) as well as advice given for ‘recently diagnosed’ families (6c).

## Data Availability

Datasets used and/or analyzed during the current study are available from the corresponding author on reasonable request.
