# Supplementary figures and images for: Challenging behavior in mucopolysaccharidoses types I–III and day-to-day coping strategies: a cross sectional explorative study
Source: Orphanet J Rare Dis. 2020 Oct 2;15:275. doi: 10.1186/s13023-020-01548-9 (PMC7532084; doi:10.1186/s13023-020-01548-9)

Additional file 1: Flowchart

*Flow chart on how the sample size was arrived*

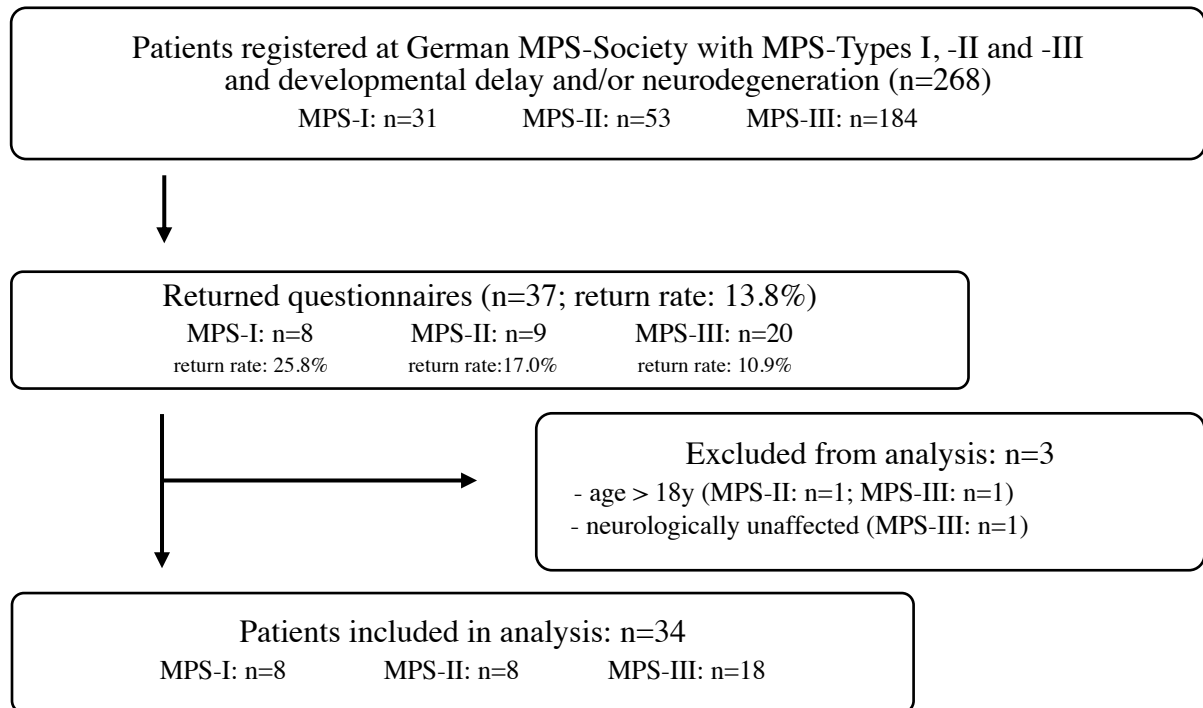

Supplement: Supplementary file 1 — Additional file 1: Flowchart. Description: flowchart on how the sample size was arrived. [file 13023_2020_1548_MOESM1_ESM.pdf]
