# Supplementary material for: Challenging behavior in mucopolysaccharidoses types I–III and day-to-day coping strategies: a cross sectional explorative study
Source: Orphanet J Rare Dis. 2020 Oct 2;15:275. doi: 10.1186/s13023-020-01548-9 (PMC7532084; doi:10.1186/s13023-020-01548-9)
Supplement: Supplementary file 2 — Additional file 2: Reported practical coping measures–categorization and perceived effectiveness. Description: Tabular presentation of reported practical coping measures against challenging behavior (2a-i), their perceived effectiveness and subsequent categorization. [file 13023_2020_1548_MOESM2_ESM.pdf]

Additional file 2: Reported practical coping measures – categorization and perceived effectiveness

2a) Measures reported against sleep disturbance

Category	Effect (M^1)	n	%	%Rep[2]	Measure	Effect (M^1)	n	%	%Rep[2]
**Distraction/Busying**	3.4	5	14.7	17.2	Watching TV	4.1	2	5.9	6.9
					Listening to music	2.8	2	5.9	6.9
					Fresh air	3.3	1	2.9	3.4
					Star projector	3.3	1	2.9	3.4
					Listening to audiobook	2.6	1	2.9	3.4
**Safety/Relief**	3.0	17	50.0	58.6	Sleeping in parent bed	2.7	11	32.4	37.9
					Bathing	1.7	5	14.7	17.2
					Pacifier	5.0	1	2.9	3.4
					Stuffed animal for chewing	4.4	1	2.9	3.4
					Calm evenings	4.1	1	2.9	3.4
					Constant repetition, same story, CD, rituals	4.0	1	2.9	3.4
					Lots of body contact	2.9	1	2.9	3.4
					Parent sleeping in child bed	2.5	1	2.9	3.4
**Frame Conditions**	3.2	24	70.6	82.8	Daytime exercise	2.7	13	38.2	44.8
					Changing type of bed (crib/nursing bed)	3.7	12	35.3	41.4
					Bedtime routine	2.6	11	32.4	37.9
					(Completely) darkening room	3.8	7	20.6	24.1
					Regular daytime routine	4.0	2	5.9	6.9
					Fixed bedtime	1.6	2	5.9	6.9
					Reduced meal-size for dinner	5.0	1	2.9	3.4
					Avoiding afternoon nap	3.9	1	2.9	3.4
					Securing bedroom	3.7	1	2.9	3.4
					Transition of exercise- and ease times	3.3	1	2.9	3.4
					Flexible bedtime	2.4	1	2.9	3.4
**Operant Conditioning**	4.2	1	2.9	3.4	Never taking child out of bed, only sitting with it	4.2	1	2.9	3.4
**Professional Therapy**	-	-	-	-	-	-	-	-	-
**Breathing Support**	4.7	2	5.9	6.9	CPAP	5.0	2	5.9	6.9
					Oxygen	5.0	1	2.9	3.4
					Inhalation therapy	2.9	1	2.9	3.4
**Medication**	3.4	14	41.2	48.3	Melatonin	2.1	8	23.5	27.6
					Pipamperone	4.2	3	8.8	10.3
					Risperidone	4.8	2	5.9	6.9

				Promethazine	4.5	2	5.9	6.9
				Guanfacine	4.9	1	2.9	3.4
				Levomepromazine	4.8	1	2.9	3.4
				Chlorale hydrate	4.4	1	2.9	3.4
				Homeopathy	4.4	1	2.9	3.4
**Total**	**3.2**	**29**	**85.3**					

)[1] M = mean perceived effectiveness as reported on visual analogue scales ranging from 0.0 (not effective) to 5.00 (very effective)
)[2] %Rep. refers to the number of questionnaires reporting strategies against sleep disturbance (n=29)

2b) Measures reported against hyperactivity

Category	Effect (M[1])	n	%	%Rep[2]	Measure	Effect (M[1])	n	%	%Rep[2]
**Distraction/Busying**	3.3	22	64.7	95.7	Exercise	3.3	16	47.1	69.6
					Busying	3.3	10	29.4	43.5
					Singing	3.4	8	23.5	34.8
					Watching TV	3.7	6	17.6	26.1
					Going for a walk	2.8	3	8.8	13.0
					Listening to music	4.1	2	5.9	8.7
					Distraction	2.8	2	5.9	8.7
					Going out in the garden	3.5	1	2.9	4.3
**Safety/Relief**	3.9	10	29.4	43.5	Seat belt	4.5	6	17.6	26.1
					Fixation in Buggy	3.7	3	8.8	13.0
					Calming child down	3.3	2	5.9	8.7
					Crib	5.0	1	2.9	4.3
					Playpen	4.7	1	2.9	4.3
					Driving in car	4.1	1	2.9	4.3
					Inner ease	2.4	1	2.9	4.3
**Frame Conditions**	3.4	14	41.2	60.9	Regular daytime routine	3.4	14	41.2	60.9
					Securing closets and roomss	4.4	1	2.9	4.3
					Locking doors	4.0	1	2.9	4.3
					Avoiding stimulus satiation	3.6	1	2.9	4.3
**Operant Conditioning**	2.0	4	11.8	17.4	Holding on child	2.0	4	11.8	17.4
**Professional Therapy**	2.5	4	11.8	17.4	Physiotherapy	3.3	2	5.9	8.7
					Music therapy	3.1	2	5.9	8.7
					Equine-assisted therapy	1.2	2	5.9	8.7
**Medication**	3.4	3	8.8	13.0	Risperidone	4.0	1	2.9	4.3
					Guanfacine	4.0	1	2.9	4.3
					Pipamperone	3.5	1	2.9	4.3
					Sertraline	2.8	1	2.9	4.3
					Homeopathy	2.5	1	2.9	4.3
**Total**	**3.4**	**23**	**67.6**						

)[1] M = mean perceived effectiveness as reported on Visual Analogue Scales ranging from 0.0 (not effective) to 5.00 (very effective)
)[2] %Rep. refers to the number of questionnaires reporting strategies against hyperactivity (n=23)

2c) Measures reported against aggression

Category	Effect (M[1])	n	%	%Rep[2]	Measure	Effect (M[1])	n	%	%Rep[2]
					Exercise	2.6	6	17.6	37.5
					Singing	3.6	5	14.7	31.3
					Painting	4.0	2	5.9	12.5
**Distraction/Busying**	3.4	12	35.3	75.0	Distraction	3.7	2	5.9	12.5
					Music	3.9	1	2.9	6.3
					Making for variety	2.2	1	2.9	6.3
					Busying	3.3	2	5.9	12.5
					Calming child down	2.7	9	26.5	56.3
					Giving attention	4.2	2	5.9	12.5
					No hecticness, give child time	4.9	1	2.9	6.3
**Safety/Relief**	3.0	12	35.3	75.0	Giving body contact	4.9	1	2.9	6.3
					Staying calm	4.8	1	2.9	6.3
					Allowing child own pace	4.8	1	2.9	6.3
					Laying child down, letting it rest	2.7	1	2.9	6.3
					Holding on child	2.3	1	2.9	6.3
**Frame Conditions**	4.0	2	5.9	12.5	Respect, love, dignity	4.9	1	2.9	6.3
					Prevenience (avoiding situation)	3.1	1	2.9	6.3
					Refusing aggression	2.4	8	23.5	50.0
**Operant Conditioning**	2.4	12	35.3	75.0	Ignoring aggression	2.4	6	17.6	37.5
					Rewarding/punishing/scolding child	2.0	4	11.8	25.0
					Eqiuine-assisted therapy	2.4	2	5.9	12.5
**Professional Therapy**	2.4	3	8.8	18.8	Music therapy	2.4	1	2.9	6.3
					Therapies	2.3	1	2.9	6.3
**Medication**	4.6	1	2.9	6.3	Promethazine	4.6	1	2.9	6.3
**Total**	**3.0**	**16**	**47.1**						

)[1] M = mean perceived effectiveness as reported on Visual Analogue Scales ranging from 0.0 (not effective) to 5.00 (very effective)
)[2] %Rep. refers to the number of questionnaires reporting strategies against aggression (n=16)

2d) Measures reported against repeated behavior

Category	Effect (M[1])	n	%	%Rep[2]	Measure	Effect (M[1])	n	%	%Rep[2]
**Distraction/Busying**	3.2	10	29.4	66.7	Busying	3.5	7	20.6	46.7
					Exercise	3.3	4	11.8	26.7
					Singing	4.1	3	8.8	20.0
					Distraction	2.5	2	5.9	13.3
					Child sitting on gym ball	2.7	1	2.9	6.7
**Safety/Relief**	3.0	2	5.9	13.3	Body contact	4.9	1	2.9	6.7
					Holding on child	1.1	1	2.9	6.7
**Frame Conditions**	4.0	11	32.4	73.3	Locking doors*	4.6	3	8.8	20.0
*Preventing behavior	3.8	9	26.5	60.0	Covering light switches*	4.8	2	5.9	13.3
					Securing Refrigerator*	3.5	2	5.9	13.3
					Taking key out of lock*	5.0	1	2.9	6.7
					Securing shutters*	5.0	1	2.9	6.7
					Turning off water supply of water-tap*	5.0	1	2.9	6.7
					Rotatable doorknobs*	5.0	1	2.9	6.7
					Securing appartment*	5.0	1	2.9	6.7
					Hazard-free room	5.0	1	2.9	6.7
					1:1-supervision	4.9	1	2.9	6.7
					Regular daytime routine	4.8	1	2.9	6.7
					Completely darkening room	4.1	1	2.9	6.7
					Hiding smartphone*	3.7	1	2.9	6.7
					Removing objects	2.5	1	2.9	6.7
					Grid to siblings rooms*	2.4	1	2.9	6.7
					Turning off electricity*	0.1	1	2.9	6.7
**Operant Conditioning**	1.2	2	5.9	13.3	Talking at child	2.4	1	2.9	6.7
					Undoing child's action (e.g. lights on)	0.0	1	2.9	6.7
**Professional Therapy**	3.9	3	8.8	20.0	Physiotherapy	4.6	1	2.9	6.7
					Attachment-Therapy	4.2	1	2.9	6.7
					Equine-assisted Therapy	2.8	1	2.9	6.7
**Medication**	4.2	1	2.9	6.7	Homeopathy	4.2	1	2.9	6.7
					Promethazine	4.2	1	2.9	6.7
**Total**	**3.5**	**15**	**44.1**						

)[1] M = mean perceived effectiveness as reported on Visual Analogue Scales ranging from 0.0 (not effective) to 5.00 (very effective)
)[2] %Rep. refers to the number of questionnaires reporting strategies against repeated behavior (n=15)

2e) Measures reported against agitation

Category	Effect (M[1])	n	%	%Rep[2]	Measure	Effect (M[1])	n	%	%Rep[2]
**Distraction/Busying**	2.4	1	2.9	50.0	Exercise	2.4	1	2.9	50.0
**Safety/Relief**	2.7	2	5.9	100	Putting child in rehab buggy	4.6	1	2.9	50.0
					Pacifier	2.4	1	2.9	50.0
					Fixation	1.8	1	2.9	50.0
**Frame Conditions**	-	-	-	-	-	-	-	-	-
**Operant Conditioning**	-	-	-	-	-	-	-	-	-
**Professional Therapy**	-	-	-	-	-	-	-	-	-
**Medication**	-	-	-	-	-	-	-	-	-
**Total**	**2.5**	**2**	**5.9**						

** Agitation was not directly prompted for coping measures*
)[1] M = mean perceived effectiveness as reported on Visual Analogue Scales ranging from 0.0 (not effective) to 5.00 (very effective)
)[2] %Rep. refers to the number of questionnaires reporting strategies against agitation (n=2)

*2f) Measures reported against unusual affect**

Category	Effect (M[1])	n	%	%Rep[2]	Measure	Effect (M[1])	n	%	%Rep[2]
**Distraction/Busying**	1.7	2	5.9	50	Singing	1.7	2	5.9	50.0
					Watching TV	0.0	1	2.9	25.0
**Safety/Relief**	2.6	1	2.9	25	Showing empathy	2.6	1	2.9	25.0
**Frame Conditions**	2.0	1	2.9	25	Searching for potential soucre of pain	2.0	1	2.9	25.0
**Operant Conditioning**	2.6	2	5.9	50	Ignoring behavior	2.7	1	2.9	25.0
					Assertive talking at child	2.5	1	2.9	25.0
**Proffessional Therapy**	**-**	**-**	**-**	**-**	-	-	-	-	-
**Medication**	3.6	1	3.6	25	Medication (not specified)	3,8	1	2.9	25.0
					Levomepromazine	3,3	1	2.9	25.0
**Total**	**2.2**	**4**	**11.8**						

** Unusual affect was not directly prompted for coping measures*
)[1] M = mean perceived effectiveness as reported on Visual Analogue Scales ranging from 0.0 (not effective) to 5.00 (very effective)
)[2] %Rep. refers to the number of questionnaires reporting strategies against unusual affect (n=4)

*2g) Measures reported against orality**

Category	Effect (M[1])	n	%	%Rep[2]	Measure	Effect (M[1])	n	%	%Rep[2]
**Distraction/Busying**	3.4	4	11.8	80.0	Biting rings	3.8	3	8.8	60.0
					Giving food	3.0	1	2.9	20.0
					Distraction	1.1	1	2.9	20.0
**Safety/Relief**	3.1	1	2.9	20.0	Pacifier	3.1	1	2.9	20.0
**Frame Conditions**	3.0	2	5.8	40.0	Shutting away dangerous objects	3.5	1	2.9	20.0
					Removing objects, spartan furnishing	2.5	1	2.9	20.0
**Operant Conditioning**	2.3	1	2.9	20.0	Reward and punishment	2.3	1	2.9	20.0
**Proffessional Therapy**	-	-	-	-	-	-	-	-	-
**Medication**	-	-	-	-	-	-	-	-	-
**Total**	**3.1**	**5**	**14.7**						

** Orality was not directly prompted for coping measures*
)[1] M = mean perceived effectiveness as reported on Visual Analogue Scales ranging from 0.0 (not effective) to 5.00 (very effective)
)[2] %Rep. refers to the number of questionnaires reporting strategies against orality (n=5)

2h) Other openly reported behavioral symptoms and regarding measures

Symptom	Symptom reported (n)	Measure	Effect (M[1])	n	%	%Rep[2]
Child can not be alone, always needs busying	1	-	-	-	-	-
Child can not detect dangerous situations	2	-	-	-	-	-
Cognitive deficits	1	Exercise	4.3	1	2.9	100
		Making child laugh	4.3	1	2.9	100
		Repetition of games	2.2	1	2.9	100
		Supporting and challenging child	2.2	1	2.9	100
Destructive behavior	1	-	-	-	-	-
Fearfulness	1	Being with child	2.5	1	2.9	100
Child is insistent on windows being closed	1	-	-	-	-	-
Non-cooperative when being fed	1	Fixation of childs hands	4.0	1	2.9	100
Child notices ist differentness	1	Accepting child as it is	4.8	1	2.9	100
		Praise/appreciation	4.8	1	2.9	100
		Intense eye- and bodycontact	3.5	1	2.9	100
Obstinacy	1	Loving encouragement	0.9	1	2.9	100
		Reward and punishment	0.4	1	2.9	100
Panic attacks	1	Repeated introduction into situations	3.7	1	2.9	100
Child runs away	2	Locking doors and windows	5.0	2	5.9	100
		1:1-supervision	4.9	1	2.9	50.0
		Dayly routine	4.9	1	2.9	50.0
		Fixation	4.1	1	2.9	50.0
Child scratches itself	2	covering fingers and scratching-spots (patches)	3.8	1	2.9	100
		Cutting nails rather short	2.9	1	2.9	100
		Keeping childs hands busy	2.6	1	2.9	100
Sensivity to noise	1	Avoiding noisy situations	5.0	1	2.9	100
Shyness	1	Recurring activities	3.6	1	2.9	100
		Familiar groups	2.7	1	2.9	100
		Encouragement	2.5	1	2.9	100
Speech disorder	1	Questioning child	2.4	1	2.9	100
		Sign-supported communication	2.1	1	2.9	100

)[1] M = mean perceived effectiveness as reported on Visual Analogue Scales ranging from 0.0 (not effective) to 5.00 (very effective)
)[2] %Rep. refers to the number of questionnaires reporting strategies against the particular symptom

2i) Openly reported somatic symptoms and regarding measures:

Symptom	Symptom reported (n)	Measure	Effect (M[1])	n	%	%Rep[2]
Abesences	1	Valproate	4.5	1	2.9	100
		Putting child in bed	3.4	1	2.9	100
		Going for walk in Buggy	3.2	1	2.9	100
Child can not sit without aid	1	Stable seat	3.5	1	2.9	100
		Assistant	4.2	1	2.9	100
Diarrhea	1	Probiotics	2.5	1	2.9	100
Dysphagia	1	-	-	-	-	-
Incontinence	2	Bowel training	2.5	1	2.9	100
		Reward	3.2	1	2.9	100
Multifocal seizures	1	Medication	0.1	1	2.9	100
		1:1 supervision	0.1	1	2.9	100
Obstipation	1	Laxantives	2.3	1	2.9	100
		Clyster	0.0	1	2.9	100
		Oral hydration	0.0	1	2.9	100
Walking problems	1	-	-	-	-	-

)[1] M = mean perceived effectiveness as reported on Visual Analogue Scales ranging from 0.0 (not effective) to 5.00 (very effective)
)[2] %Rep. refers to the number of questionnaires reporting strategies against the particular symptom
