# Supplementary material for: Challenging behavior in mucopolysaccharidoses types I–III and day-to-day coping strategies: a cross sectional explorative study
Source: Orphanet J Rare Dis. 2020 Oct 2;15:275. doi: 10.1186/s13023-020-01548-9 (PMC7532084; doi:10.1186/s13023-020-01548-9)
Supplement: Supplementary file 3 — Additional file 3: Ability Score–calculation and results. Tabular presentation of calculation of the Ability Score (3a) and results in individual MPS-types (3b). [file 13023_2020_1548_MOESM3_ESM.pdf]

Additional file 3: Ability Score – calculation and results

3a) Ability Score: Calculation

Best possible child ability in		Possible points	Child (sub-)score
Mobility	Walks free	(2)	
	Needs walking aid	(1)	______
	Wheelchair bound	(0)	
Speech	Not restricted	(2)	
	Some words	(1)	______
	No speech	(0)	
Feeding	Independent	(2)	
	Needs feeding	(1)	______
	Gastrostomy	(0)	

Total: Ability Score = ______

3b) Ability Score: Results

	**MPS-I** (n=8)	**MPS-II** (n=8)	**MPS-III** (n=18)	**All** (n=34)			Post Hoc		
	M ± SD	M ± SD	M ± SD	M ± SD	H	p	MPS	U	p
Mobility	1.5 ± 0.5	1.6 ± 0.7	1.2 ± 0.9	1.4 ± 0.8	1.54	.454			
							I:II	-	NS
Speech	1.9 ± 0.4	1.3 ± 0.9	0.7 ± 0.8	1.1 ± 0.9	11.35	.003	I:III	16.5	.001
							II:III	-	NS
Feeding	2.0 ± 0.0	1.6 ± 0.7	1.6 ± 0.6	1.7 ± 0.6	3.94	.150			
							I:II	-	NS
**Ability Score**	**5.4 ± 0.5**	**4.5 ± 2.1**	**3.4 ± 1.9**	**4.2 ± 1.9**	7.45	.024	I:III	22.5	.007
							II:III	-	NS
