# Supplementary material for: Challenging behavior in mucopolysaccharidoses types I–III and day-to-day coping strategies: a cross sectional explorative study
Source: Orphanet J Rare Dis. 2020 Oct 2;15:275. doi: 10.1186/s13023-020-01548-9 (PMC7532084; doi:10.1186/s13023-020-01548-9)
Supplement: Supplementary file 4 — Additional file 4: Reported symptoms–frequency, parent- and child stress. Presentation of reported symptoms in individual MPS-types in 3 tables: frequency (4a), parent stress (4b) and child stress (4c). [file 13023_2020_1548_MOESM4_ESM.pdf]

Additional file 4: Reported symptoms – frequency, parent- and child stress

4a) Reported symptom frequency[1]

	**MPS I** (n=8) n; M ± SD	**MPS II** (n=8) n; M ± SD	**MPS III** (n=18) n; M ± SD	**all** (n=34) n; M ± SD	H	p	Post Hoc	U	p
sleep disturbance	8; 1.4 ± 2.0	8; 2.3 ± 1.9	18; 2.5 ± 2.0	34; 2.2 ± 2.0	1.75	.416			
hyperactivity	8; 0.4 ± 0.8	8; 3.5 ± 1.7	18; 2.2 ± 2.1	34; 2.1 ± 2.0	7.57	.023	I:II	6.0	.004
							I:III	-	NS
							II:III	-	NS
agitation	8; 0.5 ± 0.9	8; 3.2 ± 2.1	18; 2.9 ± 1.7	34; 2.4 ± 1.9	10.45	.005	I:II	9.0	.014
							I:III	16.0	.001
							II:III	-	NS
aggression	8; 1.0 ± 1.4	7; 1.7 ± 1.7	16; 1.0 ± 1.4	31; 1.2 ± 1.4	0.32	.851			
repeated behavior	8; 0.0 ± 0.0	8; 2.1 ± 2.0	18; 2.0 ± 1.9	34; 1.6 ± 1.9	8.89	.012	I:II	11.5	.023
							I:III	21.0	.002
							II:III	-	NS
unusual affect	8; 0.7 ± 1.3	8; 2.1 ± 2.3	18; 1.9 ± 1.6	34; 1.7 ± 1.8	2.46	.292			
apathy	7; 0.3 ± 0.7	7; 1.5 ± 2.3	17; 1.6 ± 2.0	31; 1.3 ± 1.9	2.87	.238			
orality*	-	3; 5.0 ± 0.0	4; 4.8 ± 0.4	7; 4.9 ± 0.3					
Average of reported symptoms	8; 0.7 ± 0.5	8; 2.5 ± 1.4	18; 2.2 ± 1.1	34; 1.9 ±1.3	10.97	.004	I:II	8.0	.010
							I:III	15.0	.001
							II:III	-	NS

)[1] Frequency as reported on Visual Analogue Scales with range 0.0 (low) to 5.0 (high)
** Orality was not directly prompted, values may be overestimations (see Discussion)*

4b) Reported parent stress[1] in individuals with relevant (at least moderate) symptom frequency

	**MPS I** (n=8)	**MPS II** (n=8)	**MPS III** (n=18)	**all** (n=34)
	n;<br>M ± SD	n;<br>M ± SD	n;<br>M ± SD	n;<br>M ± SD
sleep disturbance	3;<br>2.6 ± 2.2	5;<br>3.8 ± 0.9	11;<br>3.8 ± 1.5	19;<br>3.6 ± 1.5
hyperactivity	1;<br>0.2 ± /	7;<br>2.5 ± 1.4	10;<br>4.0 ± 1.5	18;<br>3.2 ± 1.7
agitation	1;<br>2.7 ± /	6;<br>3.2 ± 1.5	13;<br>3.7 ± 1.6	20;<br>3.5 ± 1.5
aggression	3;<br>3.0 ± 2.5	4;<br>3.9 ± 1.1	5;<br>3.4 ± 1.3	12;<br>3.5 ± 1.5
repeated behavior	-	5;<br>2.0 ± 1.6	9;<br>3.0 ± 2.2	14;<br>2.7 ± 2.0
unusual affect	1;<br>5.0 ± /	4;<br>2.4 ± 0.2	11;<br>3.1 ± 1.6	16;<br>3.0 ± 1.5
apathy	1;<br>1.4 ± /	2;<br>0.1 ± .0	6;<br>3.7 ± 0.9	9;<br>2.6 ± 1.8
orality*	-	3;<br>3.9 ± 1.5	4;<br>4.5 ± 0.6	7;<br>4.2 ± 1.0

Comparison of subgroups not displayed due to variation and extreme inequality of subgroup sizes
)[1] Parent stress as reported on Visual Analogue Scales with range 0.0 (low) to 5.0 (high)
** Orality was not directly prompted, values may be overestimations (see Discussion)*

4c) Reported child stress[1] in individuals with relevant (at least moderate) symptom frequency

	**MPS I** (n=8)	**MPS II** (n=8)	**MPS III** (n=18)	**all** (n=34)
	n;<br>M ± SD	n;<br>M ± SD	n;<br>M ± SD	n;<br>M ± SD
sleep disturbance	3;<br>3.2 ± 0.9	5;<br>2.6 ± 1.4	11;<br>3.5 ± 1.5	19;<br>3.2 ± 1.4
hyperactivity	1;<br>0.4 ± /	7;<br>1.5 ± 1.3	10;<br>2.8 ± 1.7	18;<br>2.1 ± 1.7
agitation	1;<br>4.0 ± /	6;<br>2.0 ± 1.8	13;<br>2.3 ± 1.8	20;<br>2.3 ± 1.8
aggression	2;<br>3.2 ± 1.0	4;<br>1.4 ± 2.2	5;<br>2.2 ± 0.9	11;<br>2.1 ± 1.5
repeated behavior	–	5;<br>0.8 ± 0.9	9;<br>0.6 ± 1.0	14;<br>0.7 ± 1.0
unusual affect	1;<br>4.8 ± /	4;<br>0.9 ± 1.1	11;<br>2.5 ± 1.6	16;<br>2.2 ± 1.7
apathy	1;<br>0.3 ± /	2;<br>0.1 ± 0.0	5;<br>1.8 ± 1.9	8;<br>1.2 ± 1.7
orality*	–	3;<br>0.4 ± 1.5	4;<br>2.0 ± 1.9	7;<br>1.3 ± 1.9

Comparison of subgroups not displayed due to variation and extreme inequality of subgroup sizes
)[1] Child stress as reported on Visual Analogue Scales with range 0.0 (low) to 5.0 (high)
** Orality was not directly prompted, values may be overestimations (see Discussion)*
