# Supplementary material for: Challenging behavior in mucopolysaccharidoses types I–III and day-to-day coping strategies: a cross sectional explorative study
Source: Orphanet J Rare Dis. 2020 Oct 2;15:275. doi: 10.1186/s13023-020-01548-9 (PMC7532084; doi:10.1186/s13023-020-01548-9)
Supplement: Supplementary file 5 — Additional file 5: Reported medication therapy. Tabular presentation of reported Drug therapy against challenging behavior and its perceived effectiveness, as well as comparison of total effectiveness of medication- and non-medication-measures. [file 13023_2020_1548_MOESM5_ESM.pdf]

Additional file 5: Reported medication therapy

*Medication reported against challenging behavior*

| | **Sleep disturbance** | | **Hyperactivity** | | **Aggression** | | **Repeated behavior** | | **Unusual Affect** | |
|---|---|---|---|---|---|---|---|---|---|---|
| | n | Effect (M) | n | Effect (M) | n | Effect (M) | n | Effect (M) | n | Effect (M) |
| **Melatonin** | 8 | 2.1 | | | | | | | | |
| **Antipsychotics** | 8 | 4.5 | | | | | | | | |
| Risperidone | 2 | 4.8 | 1 | 4.0 | | | | | | |
| Levomepromazine | 1 | 4.8 | | | | | | | 1 | 3.3 |
| Pipamperone | 3 | 4.2 | 1 | 3.5 | | | | | | |
| Promethazine | 2 | 4.5 | | | 1 | 4.6 | 1 | 4.2 | | |
| **Antisymtathetics** | | | | | | | | | | |
| Guanfacine | 1 | 4.9 | 1 | 4.0 | | | | | | |
| **Chloral Hydrate** | 1 | 4.4 | | | | | | | | |
| **Antidepressants** | | | | | | | | | | |
| Sertraline | | | 1 | 2.8 | | | | | | |
| **Homeopathy** | 1 | 4.4 | 1 | 2.5 | | | 1 | 4.2 | | |
| **Not specified** | | | | | | | | | 1 | 3.8 |
| **Total** | 14 | 3.4 | 3 | 3.4 | 1 | 4.6 | 2 | 4.2 | 1 | 3.6 |
| Comparison: **Non-medication-therapies (total)** | 28 | 3.2 | 23 | 3.4 | 16 | 3.0 | 15 | 3.5 | 4 | 2.0 |

*Drug use reported in questionnaires (n) and their perceived effectiveness as rated on Visual Analogue Scales with range 0.00 (low) to 5.00 (high)*
