# Supplementary material for: Challenging behavior in mucopolysaccharidoses types I–III and day-to-day coping strategies: a cross sectional explorative study
Source: Orphanet J Rare Dis. 2020 Oct 2;15:275. doi: 10.1186/s13023-020-01548-9 (PMC7532084; doi:10.1186/s13023-020-01548-9)
Supplement: Supplementary file 6 — Additional file 6: Coping the disorder. Description: Tabular presentation of reported acceptance of illness and personal coping strategies (6a), the disorders impact on family relationships and strategies for strengthening them (6b) as well as advice given for ‘recently diagnosed’ families (6c). [file 13023_2020_1548_MOESM6_ESM.pdf]

Additional file 6: Coping the disorder

6a) Acceptance of illness and personal strategies for coping the disorder

	**Strategy**	**Value[1]/ Effect (M[1])**	**n**	**%**	**%Rep.[2]**
Acceptance of illness		2.7	33	100	-
Personal coping – reported strategies	Distraction/timeouts outside the family	3.3	28	84.8	87.5
	Communicating with Friends/relatives/acquaintances	3.4	26	78.8	81.3
	Sports	2.6	19	57.6	59.4
	Psychotherapy	1.5	14	42.2	43.8
	Homeopathy	0.0	8	24.2	25.0
	Communicating with other MPS-families*	4.2	4	12.1	12.5
	Hospice stays*	4.5	3	9.1	9.4
	Self-information about MPS*	4.1	3	9.1	9.4
	Working*	4.6	2	6.1	6.3
	Conforming daily routine to child*	5	1	3.0	3.1
	Writing diary*	4.6	1	3.0	3.1
	Strengthening family spirit*	4.4	1	3.0	3.1
	Seeing the child, not the disorder*	4.4	1	3.0	3.1
	Music*	4.1	1	3.0	3.1
	Open/natural way of dealing with MPS*	1.3	1	3.0	3.1
Importance of...	.. time-outs for oneself	4.5	27	81.8	-
	.. networking with other MPS-families	3.6	31	93.9	-

)[1] Acceptance, effectiveness and importance as reported on VAS with range 0.0 (low) to 5.0 (high)
)[2] %Rep refers to the number of participants which have reported personal coping strategies (n=32)
** coping strategy was openly reported*

6b) Impact of the disorder on family relationships and strategies for strengthening them

	Strategy	Value[1]/ Effect (M[2])	n	%	%Rep.[3]
Impact of disorder on relationship to…	.. partner	-0.2	31	93.9	-
	(Parents seperated/got divorced	-	6	18.2	-)
	.. healthy siblings	-0.4	20	60.6	-
	.. extended family	+0.19	32	97.0	-
Interfamilial Coping	Mutual support in child care	3.9	24	72.7	92.3
	Joint excursions	4.0	20	60.6	76.9
	Creation of free space for partnership*	4.0	8	24.2	30.8
	Psychotherapy	2.1	7	21.2	26.9
	Open communication*	3.5	3	9.1	11.5
	Timeouts alone*	4.3	3	9.1	11.5
	Homeopathy	0.0	3	9.1	11.5
	Excursions without MPS-Child*	5.0	2	6.1	7.7
	Excursions with MPS-Child*	4.2	2	6.1	7.7
	Taking time for each family member*	2.3	2	6.1	7.7
	Hospice stays*	4.2	1	3.0	3.8
	Releasing siblings from their disorder-related position*	2.4	1	3.0	3.8

)[1] Impact as reported on Visual Analogue Scales with range -2.5 (worsened) to +2.5 (bettered)
)[2] Effectiveness as reported on Visual Analogue Scales with range 0.0 (low) to 5.0 (high)
)[3] %Rep refers to the number of participants which have reported interfamilial coping strategies (n=26)
** coping strategy was openly reported*

6c) Advice for 'recently diagnosed' families

		n	%	%Rep.[1]
Advice	Networking with other affected Families	17	51.5	54.8
	Acceptance of illness	13	39.4	41.9
	Activating resources/consulting specialists	13	39.4	41.9
	Contacting the child	7	21.2	22.6
	Encouragement for hope/coping advice	7	21.2	22.6
	Communication and fostering family functioning	6	18.2	19.4
	Consulting psychotherapists/psychiatrist	5	15.2	16.1
	Other advice	3	9.1	9.7

)[1] %Rep refers to the number of participants which have reported advice (n=31)
